# The effects of the *Lactobacillus acidophilus ATCC 4356* on the oxidative stress of reproductive system in diabetic male rats

**DOI:** 10.18502/ijrm.v17i7.4861

**Published:** 2019-07-31

**Authors:** Seddigheh Sheikh Hosseini, Ali Gol, Moje Khaleghi

**Affiliations:** Department of Biology, Faculty of Science, University of Shahid Bahonar, Kerman, Iran.

**Keywords:** Diabetes mellitus, Oxidative stress, Rats, Reproduction, Lactobacillus acidophilus.

## Abstract

**Background:**

**Background:** Oxidative stress plays an important role in the development of diabetic complications.

**Objective:**

This study evaluated the impact of pre- and post-treatment with *Lactobacillus acidophilus ATCC 4356* on the oxidant and anti-oxidant factors of testis and epididymis in streptozotocin-induced diabetic rats.

**Materials and Methods:**

Thirty male Wistar rats (10 wk old) weighing 220-230 g. were divided into five groups (n = 6/ each): 1- normal group, 2- normal *lactobacillus* group, 3- diabetic group, 4- diabetic + *lactobacillus* before (DLB) group, and 5- diabetic + *lactobacillus after* (DLA) group. The normal and diabetic groups received daily 1 mL normal saline for 6 wk. Normal *lactobacillus* group received daily *L. acidophilus* for 6 wk. Group DLB received daily *L. acidophilus* for 2 wk before diabetes and for 4 wk after diabetes. Group DLA received daily 1 mL normal saline for 2 wk before diabetes and *L. acidophilus* for 4 wk after diabetes. The dose of *L. acidophilus* was 1 × 10${}^{9}$ CFU/mL.

**Results:**

The administration of *L. acidophilus* worsened blood glucose level and reduced the levels of Malondialdehyde (p ≤ 0.0001) and Hydrogen peroxide (p ≤ 0.0001) and, Catalase and Glutathione peroxidase activity increased in the testis. In epididymis, Glutathione peroxidase and Catalase (p = 0.013) activity increased and Hydrogen peroxide concentration reduced, while Malondialdehyde concentration did not show any changes compared to the diabetic rats. Also, there was no significant difference between DLB and DLA groups, in these markers.

**Conclusion:**

Data obtained suggests that *L. acidophilus* has anti-oxidant effects on the testis and sometime in the epididymis in diabetic rats.

## 1. Introduction

"Diabetes Mellitus is a complex metabolic disorder that is characterized by hyperglycemia resulting from defects in insulin secretion, insulin action, or both, which is associated with severe impairment in the metabolism of carbohydrates, fats and proteins” (1). ”Diabetes mellitus is a global epidemic disease that currently affect over 400 million adults worldwide, and is predicted to rise to over 600 million in 2040” (2).

One of the structures that is affected by the deleterious effects of diabetes is the male reproductive system; these complications include a decrease in testosterone level, dwindle of sexual accessory gland and decrease of desire and sexual behaviors (3, 4). Diabetic disorder also affects spermatogenesis and many studies have pointed it to reduce the sperm motility, counts, and increase abnormal sperm morphology in these diabetic patients (5, 6). Oxidative stress (OS) has been reported to play an important role in the development of diabetic complications (7). Increasing the concentration of free radicals in diabetes can reduce the function of Leydig cells and decrease testosterone production. Therefore, fertility capability decrease in diabetic individuals (8).

“It is indicated that in hyperglycemia, oxidative stress (OS) is seen due to the excessive production of reactive oxygen species (ROS) and decreased efficiency of anti-oxidant enzyme defenses” (9, 10). In the tissues of diabetic rats, Malondialdehyde (MDA) in high concentrations is considered as an index of lipid peroxidation and OS (11). Catalase (CAT), which is a highly reactive enzyme, reacts with H${}_{2}$O${}_{2}$ to form water and molecular oxygen, and also with hydrogen donors such as methanol, ethanol, formic acid, or phenols (12). Glutathione peroxidase (GPX) uses GSH to catalyze the reduction of a number of hydroperoxides (ROOH and H${}_{2}$O${}_{2}$), therefore it protects mammalian cells against oxidative damage and, reduces, among others, cellular lipid hydroperoxides (13). ”Stability and capacity of the antioxidant defense against ROS during chronic diabetes plays an important role in the outcome of long term complications caused by ROS” (14). ”So, antioxidants can be useful in the treatment of male infertility as reported” (10).

Probiotics are live microorganisms which, when administered in adequate amounts, have beneficial effects for the host through various mechanisms (15). Different genera of bacteria have been introduced as probiotics of which the most common ones are the* Lactobacillus *and *Bifidobacterium* (16). The beneficial effects of probiotics are to cure lactose intolerance, diarrhea, constipation, allergies, inflammatory intestine disease, irritable intestine syndrome, gastric ulcer, immune system stimulation, prevention of autoimmune diseases, reducing cholesterol and anti-cancer properties (17). Researchers have reported that some lactic acid bacteria have antioxidant properties (18).

Therefore, due to the prevalence of diabetes, its adverse effects on the male reproductive system, and also according to the beneficial effects of probiotics on the improvement of diabetes complications, in this study, we investigate the effect of post-treatment and pre-treatment with *Lactobacillus* *acidophilus (L.* *acidophilus) ATCC 4356* on the reproductive system of streptozotocin (STZ)- induced diabetic male rats.

## 2. Materials and Methods

This experimental study was accomplished in the Department of Biology, Faculty of Science, University of Shahid Bahonar, Kerman.

### Preparation of *L. acidophilus ATCC 4356*

*L. acidophilus ATCC 4356* was purchased as lyophilized powder (from Zist kavosh Iranian Co), cultured in MRS and incubated at 37°C for 24 hr under anaerobic conditions. Following that, it was centrifuged at 6000 rpm for 10 min, and after removing the supernatant, the harvested cells resuspended in sterile normal saline with final concentrations of 1 × 10${}^{9}$ CFU/ml (19).

### Experimental design

STZ (sigma, 60/mg body weight) (20) was used to induce diabetes. STZ Freshly prepared (dissolved in cold normal saline) was administered intraperitoneally to the rats. “Three days after STZ injection, fasting blood glucose levels measured using a Medisense Optium glucometer. Rats with blood glucose levels higher than 300 mg/dl were considered diabetics. Thirty rats were divided into five groups (n = 6)”:

• Group (N): Normal rats: Animals received daily 1 mL normal saline for 6 wk by gavage.
• Group (NL): Normal Lactobacillus: Animals received daily 1 × 10${}^{9}$ CFU/ml *L. acidophilus ATCC 4356* for 6 wk by gavage.
• Group (D): Diabetic rats: Animals received daily 1 mL normal saline for 2 wk before and 4 wk after diabetes by gavage.
• Group (DLB): diabetic+ lactobacillus before rats: Animals received daily 1 × 10${}^{9}$ CFU/ml *L. acidophilus ATCC 4356* for 2 wk before diabetes and 1 × 10${}^{9}$ CFU/ml *L. acidophilus ATCC 4356 *for 4 wk after diabetes by gavage.
• Group (DLA): diabetic+ lactobacillus after rats: Animals received daily 1 mL normal saline for 2 wk before diabetes and 1 × 10${}^{9}$ CFU/mL *L. acidophilus ATCC 4356 *for 4 wk after diabetes by gavage.

The animals were scarified on the 42nd days (21) of the experiment first deeply anesthetized with CO${}_{2}$, and then assassinated by giyotin. The testis and epididymis were dissected out and weighted. Then, they were frozen and prepared for oxidant and antioxidant assays. Animal rights were considered during all procedures.

### Catalase (CAT) and Glutathione peroxidase (GP${}_{X}$) assay 

Tissues were homogenized in 50 mM phosphate buffer (pH = 7.4). The homogenate was centrifuged at 10000 rpm for 10 min at 4°C. CAT activity was measured by the method of Aebi (22). To a cuvette containing 1.5 ml of CAT mixture (H${}_{2}$O${}_{2}$+50 mM phosphate buffer), the 100μl tissue supernatant was added. The reaction was started by the decomposition of H2O2 and CAT activity was measured using spectrophotometer at 240 nm. GP${}_{X}$ activity of tissues was measured by the method of Plewa and colleagues (23). To a cuvette containing 2.5 ml of GP${}_{X}$ mixture (H${}_{2}$O${}_{2}$ + 50 mM phosphate buffer+ guaiacol), 20μl of tissue supernatant was added. The reaction was started by the oxidation of guaiacol and GP${}_{X}$ activity was measured using spectrophotometer 470 nm.

### Malondialdehyde (MDA) assay 

Thiobarbituric Acid Reactive Substances (TBARS) level, measured as an index of MDA production and hence lipid peroxidation, were assessed in the tissues by the method of Heath and Packer (24). In brief, tissue supernatant (1 ml) was added to test tubes containing 4 ml of TCA (Trichloroacetic acid) 20% containing TBA (Thiobarbituric acid) 0.5% and the reaction mixture was heated at 95°C for 30 min and after cooling, centrifuged at 10000 gr for 10 min; MDA_TBA complex was measured using spectrophotometer at 532 nm.

### Hydrogen peroxide (H${}_{2}$O${}_{2)}$ assay 

H${}_{2}$O${}_{2}$ level measured as an index of oxidant factors, was assessed in the tissues by the method of Velikova and colleagues (25). Tissues (0.1 gr) were homogenized in 1 ml TCA (pH = 7.4). The homogenate was centrifuged at 10000 gr for 10 min at 4°C. The H${}_{2}$O${}_{2}$ concentration of tissue was measured in a cuvette containing 0.5 ml of tissue supernatant and 0.5 ml phosphate buffer 10 mM (pH = 7.4) and 1ml of Potassium Iodide 1 mM. The H${}_{2}$O${}_{2}$ concentration was measured using spectrophotometer at 390 nm.

### Ethical consideration 

The study protocol and all animal procedures were approved by the educational assistant of Faculty of Science, University of Shahid Bahonar, Kerman, Number 013, 96, 2564.

### Statistical analysis 

Data were expressed as mean ± SEM. Statistical differences between the groups were analyzed using the one-way analysis of variance (ANOVA) test and TUKEY post- test with SPSS version 16; p < 0.05 was considered significant.

## 3. Results

### Effects of *Lactobacillus acidophilus* on fasting blood glucose, body weight, testis and epididymis weight

The results of the effects of *L. acidophilus ATCC4356* at 10${}^{9}$ CFU/ml/day dose on fasting blood glucose, body weight, testes and epididymis weight of STZ-induced diabetic rats after 42 days of treatment are presented in Table I.

Glucose concentration in D, DLB and DLA groups was significantly higher compared to N and NL groups. Also DLB and DLA groups showed a significant increase in glucose concentration compared to D group.

The body weight of the D, DLB and DLA groups was significantly lower compared to that of the N and NL groups. There was no significant difference in the body weight of the D, DLB, and DLA groups.

The testis weight of the D, DLB and DLA groups were significantly lower compared to the N and NL groups.

The epididymis weight of the D, DLB, and DLA groups was significantly lower compared to that of the N and NL groups. Also, NL group showed a significant increase in the epididymis weight compared to the N group. Although N and NL groups did not show significant difference in glucose concentration, body weight, and testis weight, the NL group in these cases increased compared to N group. Also, there was no significant difference between the DLB and DLA groups in any of the aforementioned parameters.

### Effect of *Lactobacillus acidophilus* on oxidative stress (OS) in testis

**Figure 1:**
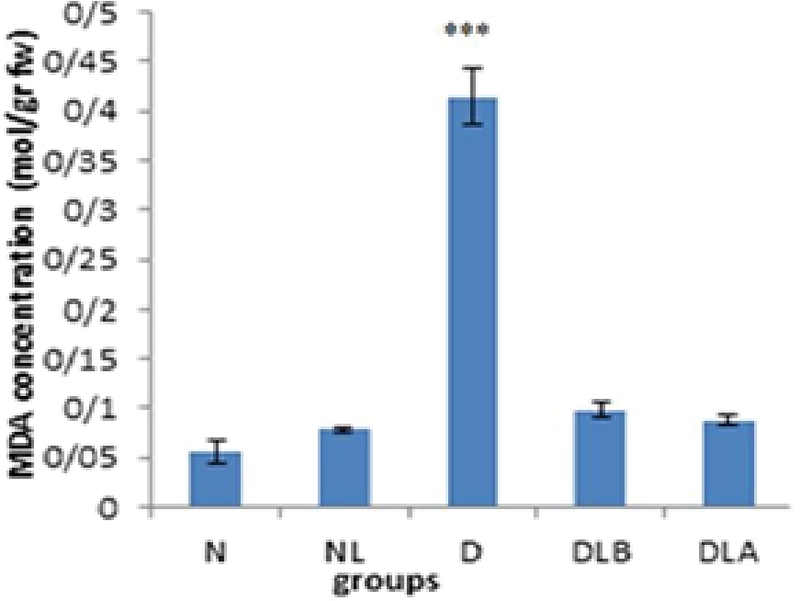
MDA concentration in testis in experimental groups. n = 6, Mean ± SEM. *** Significant difference (p ≤ 0.001) with the N, NL, DLB, and DLA groups. N: Normal group; NL: Normal lactobacillus; D: Diabetic group; DLB: Pre-treatment group; and DLA: Post-treatment group

Figure 1 shows that MDA concentration in testis in D group is significantly higher compared to the N, NL, DLB, and DLA groups.

**Figure 2:**
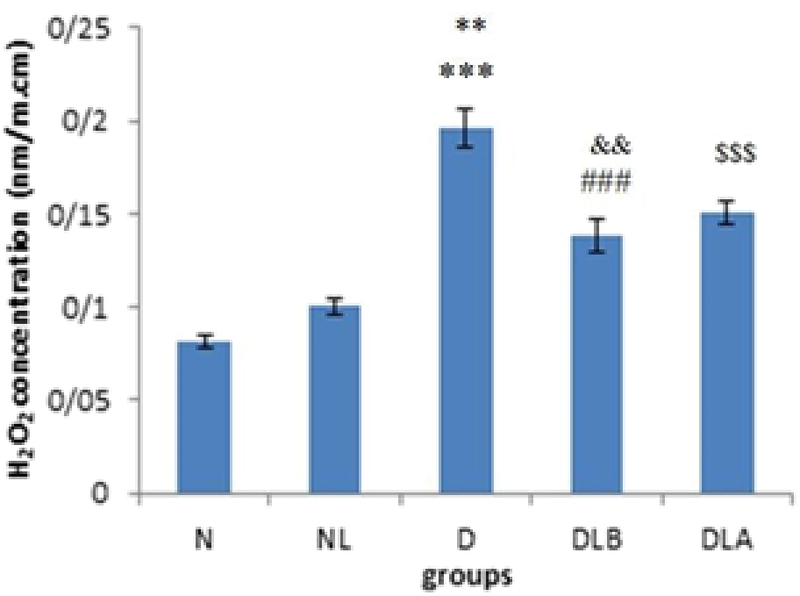
H${}_{2}$O${}_{2}$ concentration in testis in experimental groups. n = 6, Mean ± SEM. *** Significant difference with the N, NL and DLB groups (p ≤ 0.001). ** Significant difference (p ≤ 0.001) with the DLA group. $$$ Significant difference (p ≤ 0.001) with the N and NL groups. ### Significant difference (p ≤ 0.001) with the N group. && Significant difference (p = 0.008) with the NL group. N: Normal group; NL: Normal lactobacillus; D: Diabetic group; DLB: Pre-treatment group; and DLA: Post-treatment group

Figure 2 shows that H${}_{2}$O${}_{2}$ concentration in testis in D group is significantly higher compared to the N, NL, DLB, and DLA groups. Also, DLA group showed a significant increase compared to the N and NL groups, and DLB group showed a significant increase compared to the N and NL groups.

**Figure 3:**
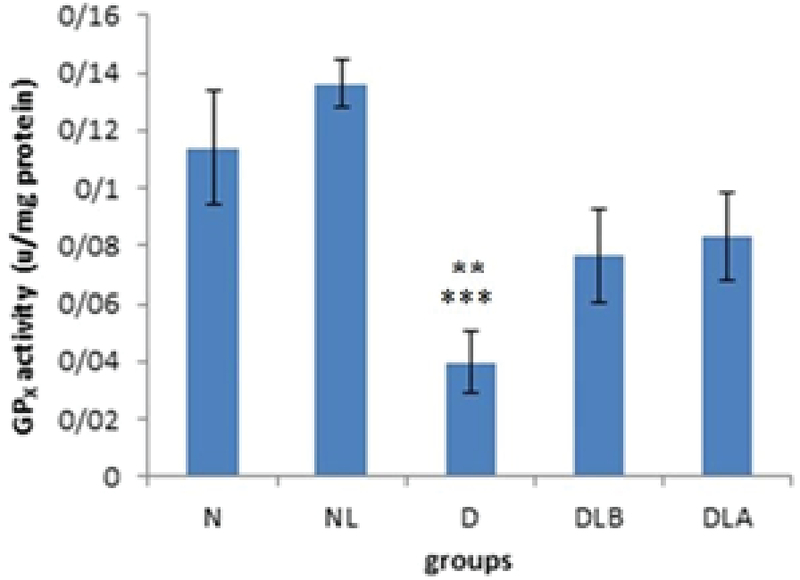
GPX activity testis in experimental groups; n = 6, Mean ± SEM. ** Significant difference (p = 0.007) with the N group. *** Significant difference (p ≤ 0.001) with the NL group. N: Normal group; NL: Normal lactobacillus; D: Diabetic group; DLB: Pre-treatment group; and DLA: Post-treatment group with the NL group

Figure 3 shows that GP${}_{X}$ activity in testis in D group is significantly lower compared to the N and NL groups. Also, while DLA and DLB groups did not show a significant difference compared to the diabetic group, they increased.

**Figure 4:**
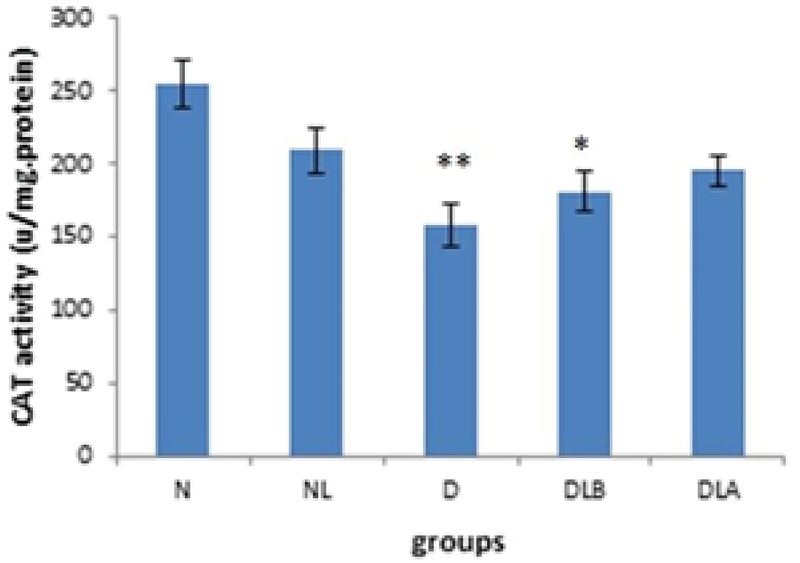
CAT activity testis in experimental groups. n = 6, Mean ± SEM. * and ** Significant difference with the N group (p = 0.011 and p ≤ 0.001, respectively). N: Normal group; NL: Normal lactobacillus; D: Diabetic group; DLB: Pre-treatment group; and DLA: Post-treatment group

Figure 4 shows that CAT activity in testis in D and DLB groups is significantly lower compared to the N group. Also, while DLA, DLB and NL groups did not show a significant difference compared to the diabetic group, they increased.

There was no significant difference between DLB and DLA groups in any of the aforementioned indicators.

### Effect of *Lactobacillus acidophilus* on oxidative stress (OS) in the epididymis 

**Figure 5:**
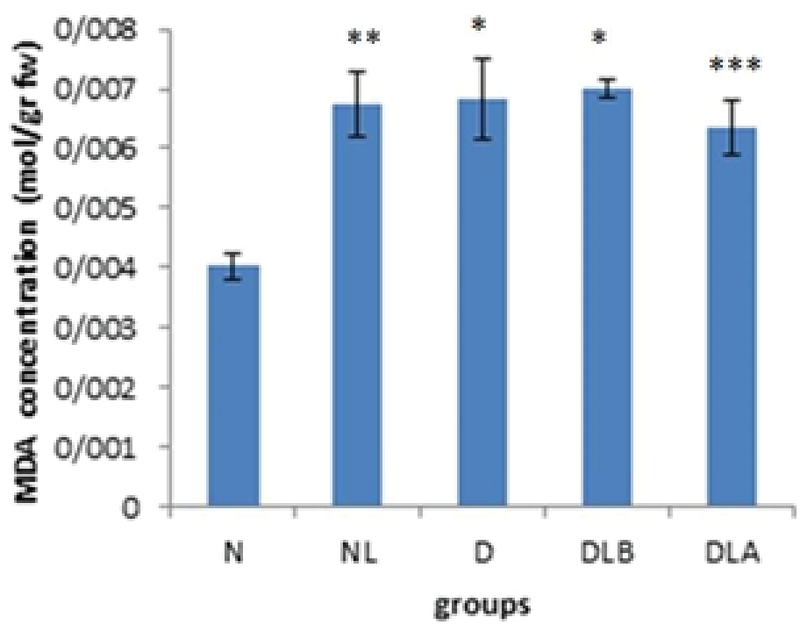
MDA concentration in epididymis in experimental groups. n = 6, Mean ± SEM. *, **, and *** Significant difference with the N group (p ≤ 0.001, p = 0.003, and p = 0.008, respectively). N: Normal group; NL: Normal lactobacillus; D: Diabetic group; DLB: Pre-treatment group; and DLA: Post-treatment group

Figure 5 shows that MDA concentration in epididymis in the NL, D, DLB and DLA groups is significantly higher compared to the N group.

**Figure 6:**
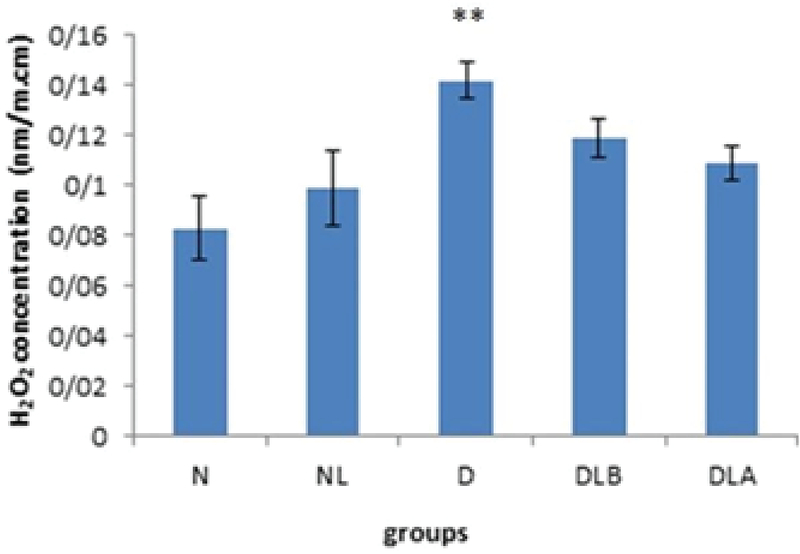
H${}_{2}$O${}_{2}$ concentration in epididymis in experimental groups. n = 6, Mean ± SEM. ** Significant difference with the N group (p = 0.004). N: Normal group; NL: Normal lactobacillus; D: Diabetic group; DLB: Pre-treatment group; and DLA: Post-treatment group

Figure 6 shows that H${}_{2}$O${}_{2}$ concentration in epididymis in the D group is significantly higher compared to the N group. Also, while the DLA, DLB and NL groups did not show a significant difference compared to the diabetic group, but they decreased.

**Figure 7:**
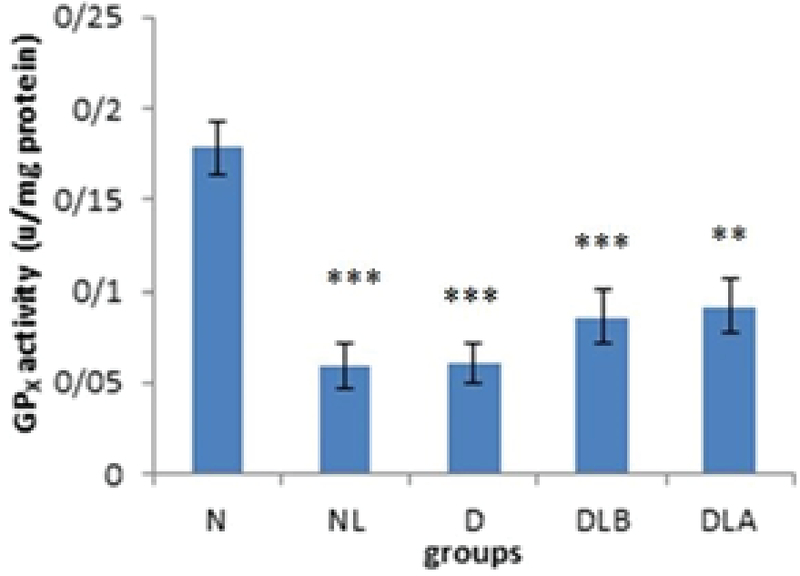
GPX activity in epididymis experimental groups. n = 6, Mean ± SEM. ** and *** Significant difference with the N group (p ≤ 0.001 and p ≤ 0.001, respectively). N: Normal group; NL: Normal lactobacillus; D: Diabetic group; DLB: Pre-treatment group; and DLA: Post-treatment group

Figure 7 shows that GP${}_{X}$ activity in testis in the DLA group and in the NL, DLB and D groups is significantly lower compared to the N group. Also, while DLA and DLB groups did not show a significant difference compared to the diabetic group, they increased.

**Figure 8:**
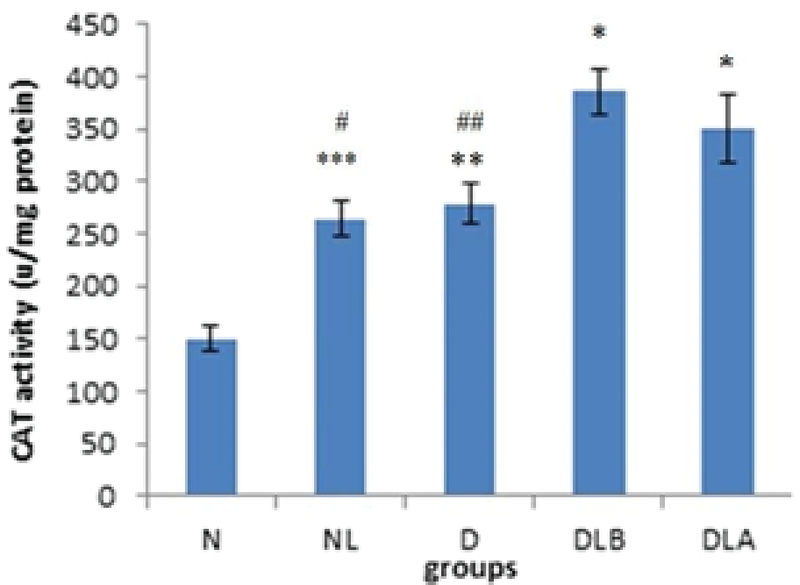
CAT activity epididymis in experimental groups. n = 6, Mean ± SEM. *, **, and *** Significant difference with the N group (p ≤ 0.001, p = 0.002, and p = 0.008, respectively). # and ##Significant difference with DLB group (p = 0.004 and p = 0.013, respectively). N: Normal group; NL: Normal lactobacillus; D: Diabetic group; DLB: Pre-treatment group; and DLA: Post-treatment group

Figure 8 shows that CAT activity in epididymis in the N group is significantly lower compared to the D, NL, DLA and DLB groups. Also, the DLB group showed a significant increase compared to the D and NL groups.

There was no significant difference between the DLB and DLA groups in any of the above indicators.

**Table 1:** Effects on fasting blood glucose, body weight, testicular and epididymis weight, in experimental groups


**Parameters**	**Group N**	**Group NL**	**Group D**	**Group DLB**	**Group DLA**
		359.14 ± 21.8	554.14 ± 25.8	573 ± 16.9
		* p ≤ 0.001	* p ≤ 0.001	* p ≤ 0.001
		# p ≤ 0.001	# p ≤ 0.001	# p ≤ 0.001
<brow>-4</erow> Glucose concentration (mg/d1)	<brow>-4</erow> 53.64 ± 3.49	<brow>-4</erow> 67.6 ± 5.3	$ p ≤ 0.001	$ p ≤ 0.001
		173.13 ± 7.4	163.63 ± 4.9	172.63 ± 6.57
		* p ≤ 0.001	* p ≤ 0.001	* p ≤ 0.001
<brow>-3</erow> Final body weight (gr)	<brow>-3</erow> 260.5 ± 3.1	<brow>-3</erow> 277 ± 3.49	# p ≤ 0.001	# p ≤ 0.001	# p ≤ 0.001
		0.952 ± 0.051	0.898 ± 0.063	0.994 ± 0.106
		* p = 0.004	* p ≤ 0.001	* p = 0.02
<brow>-3</erow> Testis weight (gr)	<brow>-3</erow> 1.27 ± 0.025	<brow>-3</erow> 1.295 ± 0.031	# p = 0.002	# p ≤ 0.001	# p = 0.01
		0. 29 ± 0.026	0. 263 ± 0.021	0.29 ± 0.028
		* p ≤ 0.001	* p ≤ 0.001	* p ≤ 0.001
<brow>-3</erow> Epididymis weight (gr)	<brow>-3</erow> 0. 513 ± 0.01	<brow>-3</erow> 0.595 ± 0.015<brow>-3</erow> * P = 0.042	# p ≤ 0.001	# p ≤ 0.001	# p ≤ 0.001
Values are mean ± SEM for six rats					
* Significant difference with N group					
# Significant difference with NL group					
$ Significant difference with D group					
N: Normal group; NL: Normal lactobacillus; D: Diabetic group; DLB: Pre-treatment group; and DLA: Post-treatment group					

## 4. Discussion

The results of the present study showed that the administration of *L. acidophilus ATCC 4356* worsened blood glucose concentration and was not effective on body, testis and epididymis weight. It improved the oxidative complications of diabetes in the testes, also in the epididymis, and improves some of the oxidative effects.

The results of this study, similar to one previous study in STZ-induced diabetic rats, showed a significant increase in the blood glucose level, and a significant reduction in the body weight, testicular, and epididymis weight compared to the normal group (26). In this study, we observed increased MDA and H${}_{2}$O${}_{2}$ level, while decreased activities of CAT and GP${}_{X}$ in diabetic group; however, CAT activity in the epididymis of the diabetic group was higher than the normal group which was Significant. Similar to these results, a previous study showed that the CAT activity in the testis of diabetic rats decreased, while in the epididymis and prostate, it increased compared to the control group (27). Also, in another study, Kakkar and colleagues showed that the CAT activity in diabetic rats increased in the heart and liver, while it decreased in the kidney (28). The difference in the CAT activity in different tissues can be due to the difference in the antioxidant capacity of different tissues and the difference in the severity of OS in them (27). ”Hyperglycemia is known to increase glycolysis, activate the intracellular sorbitol (polyol) pathway, and increase free radical formation. Glucose is oxidated into reactive ketoaldehyde and superoxide radicals. Superoxide anions are transformed into H${}_{2}$O${}_{2}$ by SOD. In the absence of breakdown by CAT or GPx, H${}_{2}$O${}_{2}$ leads to the production of reactive hydroxyl radicals. The cellular control of ROS is regulated by the antoxidative defense system, represented by enzymatic (SOD, CAT) and non-enzymatic (GSH) antioxidants. Excessive production of free radicals creates damage by binding to intracellular proteins and nucleic acids” (29).

”One previous study demonstrated that different probiotic bacteria strains could exert antioxidant capacity in different ways” (30).”Probiotics, which are capable of colonizing the intestinal tract, are reported to improve metabolic diseases such as obesity and diabetes through modulating intestinal microorganisms” (31).

The results of this study showed that despite the treatment of diabetic rats with *L. acidophilus ATCC 4356,* diabetes-induced oxidative effects in the reproductive system were improved, but they had a significant increase in the blood glucose levels compared to the diabetic group. These results were consistent with the results of a previous study that showed ”oral administration of probiotic dahi containing *L. acidophilus* and *L. casei* for 15 wk did not change the blood glucose levels in chronic hyperglycemic conditions, but reduced the OS marker thiobarbituric acid-reactive species in intestinal tissues and glycosylation of hemoglobin” (32). ”Also in another study on gestational diabetes in humans, the effects of probiotic supplementation on fasting maternal glycaemia in obese pregnant women with a Body Mass Index (BMI) of > 30 kg/m2 between 24 and 28 wk of pregnancy was investigated. A probiotic or placebo capsule was ingested daily, each probiotic capsule containing 100 mg of lyophilized *L. salivarius*. The study showed no effect of probiotic intervention during 4 wk on glycaemia” (33). ”On the other hand, one previous study has shown a decrease in blood glucose level in diabetic rats treated with different probiotics ” (34).”It is assumed that differences in the functions of several lactic acid bacteria might be the result of structural differences between species or strains” (35).

In the present study, diabetic rats treated with *L. acidophilus ATCC 4356* showed that the levels of MDA and H${}_{2}$O${}_{2}$ significantly decreased and GPx and CAT activity increased in the testis compared to the diabetic rats. Also in the epididymis, GPx and CAT activity increased and H${}_{2}$O${}_{2}$ concentration decreased compared to the diabetic rats, while MDA concentration did not show any changes compared to the diabetic rats. Also, there was no significant difference between pre and post-treatment groups, in these markers.

Similar with the results of the present study, in one study, diabetic rats fed with *L.casei, B. bifidum*, and combination group showed a highly significant increase in GPx and CAT activity in pancreatic tissue compared to the diabetic rats (34). Kumar *et al.* showed that rats receiving probiotic fermented milk had increased antioxidative enzyme activities such as CAT, SOD, and GPx compared to alloxan-induced diabetic rats during the 60 days of the experimental study (36). In another study, it was shown that the consumption of probiotic yogurt containing *L.acidophilus La5* and *Bifidobacterium lactis Bb12* for 6 wk, significantly increased erythrocyte SOD and GPx activities and significantly decreased the serum MDA concentration, in type 2 diabetic patients (37). "Another study suggested that administration of *L. acidophilus ATCC 4356* can attenuate the development of atherosclerotic lesions in ApoE-/- mice through reducing OS and inflammatory response" (38). Also, another study suggested that the administration of *L. acidophilus* maintained OS parameters from ovaries and testis (39).

The mechanisms through which *L. acidophilus* acts its antioxidant actions have not been completely understood. A study examined some of the antioxidant properties of probiotics for the following reasons: "1. Probiotics chelate metal ion. 2. Probiotics possess their own antioxidases. 3. Probiotics produce antioxidant metabolites. 4. Probiotics up-regulate antioxidase activities of the host. 5. Probiotics increase levels of antioxidant metabolites of the host. 6. Probiotics regulate signaling pathways. 7. Probiotics down-regulate activities of enzymes producing ROS. 8. Probiotics regulates intestinal microbiota" (40).

Considering the results of this study, the beneficial effect *L. acidophilus ATCC 4356 *on the reduction of diabetes complications in the reproductive system is probably through mechanisms other than reducing hyperglycemia caused by diabetes.

## 5. Conclusion

According to our findings, this is the first study to investigate the effect of probiotics on the reproductive system in diabetic rats showing that administration of *L. acidophilus ATCC 4356* improved the oxidative complications of diabetes in the testes. Also, in the epididymis, it improved some of the oxidative changes. Moreover there was no significant difference between the DLB and DLA groups.

##  Conflict of Interest

There is no conflict of interest.
